# Why People Seek Obesity Care Through Digital Rather Than In-Person Services: A Quantitative Multinational Analysis of Patients From a Large Unsubsidized Digital Obesity Provider

**DOI:** 10.7759/cureus.75603

**Published:** 2024-12-12

**Authors:** Louis A Talay, Matt Vickers

**Affiliations:** 1 Department of Medical Research, Eucalyptus, Sydney, AUS; 2 Department of Clinical Governance, Eucalyptus, Sydney, AUS

**Keywords:** access to health care, digital health technology, glp-1 receptor agonists, multidisciplinary treatment, weight loss and obesity

## Abstract

Digital modalities have been demonstrated to improve access and adherence to various chronic care services by mitigating geographical, temporal, and psychological barriers to ongoing multidisciplinary consultations, which such diseases necessitate. The net utility of medication-supported digital weight-loss services (DWLSs) has been intensely debated over the past few years due to their rapid uptake against the backdrop of the obesity epidemic. However, research on these services in real-world settings is scarce. Patients of a large multinational DWLS were emailed a four-question survey, soliciting their reasons for using the service instead of face-to-face (F2F) alternatives. Responses were collected from 1,283 patients, including 481 from the United Kingdom, 428 from Germany, and 374 from Australia. Personal discomfort in discussing weight loss in F2F settings was the most common reason for subscribing to the Eucalyptus DWLS across the full cohort (*N* = 557, 43.41%), followed by the modality’s flexibility (*N* = 441, 34.37%), patient inability to access comprehensive obesity care through a local general practitioner (GP) (*N* = 435, 33.90%), and marketing or brand awareness (*N* = 358, 27.90%). Several significant differences were observed between country, gender, ethnicity, and regular GP status across each of the subscription reasons. This study contributed another important layer to the emerging literature on DWLSs by generating preliminary quantitative evidence of their benefits to obesity care access. However, the findings also indicated that a certain number of patients may be subscribing to such services simply to access weight-loss medications rather than multidisciplinary care. To derive clearer conclusions about this concern, follow-up studies should aim to analyze health coaching engagement markers across a range of service providers.

## Introduction

Obesity is now widely acknowledged as one of the most pressing global health challenges [1]. Recent estimates indicate that approximately 2.5 billion adults are overweight, with nearly one billion classified as obese [2]. The prevalence of overweight and obesity in children and adolescents has also steadily increased over the past several decades [2]. Whereas many commentators in bygone eras considered excess weight the result of low self-discipline, modern-day experts attribute rising obesity levels to the disease’s complexity [3,4]. Specifically, they emphasize the significance of various social, economic, environmental, and biological determinants of obesity, such as marketing, food security, and neurological pathways involved in satiety [4,5,6]. As a result of these complexities, leading health organizations emphasize the need for ongoing multidisciplinary care for people living with overweight and obesity (PWOO) and discourage the use of glucose-like peptide-1 receptor agonists (GLP-1 RAs) as a stand-alone treatment for obesity [7,8]. This latter point is becoming increasingly topical as many direct-to-consumer GLP-1 RA providers have emerged in response to the unprecedented weight-loss outcomes reported in GLP-1 RA clinical trials [9,10]. However, one key factor contributing to the rise in obesity that has received comparatively less attention is the difficulty in accessing quality obesity care [11]. Unlike acute primary care settings, where access barriers are predominantly geographical, access to sustained multidisciplinary obesity care is hindered by temporal, social, and psychological (stigma-related) barriers [11,12,13]. Experts tend to agree that digital modalities help overcome these barriers in the treatment of other chronic illnesses such as diabetes and mental health disorders [14,15,16, 17]. In recent years, digital weight-loss services (DWLSs) have emerged as a potential solution to the obesity care access problem [10]. However, the literature is yet to clearly establish why increasingly large numbers of PWOO are using DWLSs rather than face-to-face (F2F) alternatives to treat their disease, and thus whether DWLSs are reducing access barriers that contribute to the obesity epidemic.

Prominent health experts have voiced concerns over the quality and safety of DWLSs [9,18]. A regular criticism is that many providers merely connect patients to previously unknown doctors who prescribe weight-loss medications based on responses to brief questionnaires, without providing follow-up care [18]. Such criticisms cannot be reasonably rebutted with the comparable safety outcomes in the control and intervention groups of GLP-1 RA and dual glucose-dependent insulinotropic polypeptide (GIP) and GLP-1 RAs efficacy trials [19,20,21,22], as these studies do not reflect real-world experiences. Moreover, they do not account for emerging evidence that weight loss through GLP-1 RAs often includes significant reductions in vital fat-free mass and tends to be regained after discontinuing the medication [22,23]. Until more is known about the long-term effects of GLP-1 RAs, all obesity programs should prioritize behavioral therapy and the involvement of multidisciplinary teams (MDTs), as recommended by major health bodies, including the World Health Organization [24]. The UK’s National Institute for Health and Care Excellence, for instance, emphasizes in its guidelines that Semaglutide, a type of GLP-1 RA, “should only be given alongside a suitably sustained programme of lifestyle interventions with multidisciplinary input” [25].

Certain DWLSs have provided early evidence of adhering to this guidance, such as Eucalyptus - a large multinational provider. Although no dedicated analyses of the Eucalyptus DWLS model have been conducted, studies on the service’s effectiveness [26,27], care continuity [28], GLP-1 RA prescribing and dispensing safety [29,30], and its patients’ adherence [31], suggest the model is suitably comprehensive. A recent qualitative study aimed to lay a foundation for research on the access utility of DWLSs by assessing the reasons patients of the Australian Eucalyptus DWLS subscribed to the service instead of F2F alternatives [11]. The analysis of 197 patient interviews identified 5 core themes behind this decision, including a failure to achieve weight-loss goals through stand-alone lifestyle interventions; marketing and brand awareness; difficulty accessing comprehensive weight-loss care through local general practitioners (GPs); more comfortable receiving weight-loss therapy through digital platforms; and valuing the flexibility of asynchronous DWLS consults.

This study aims to extend upon the findings of the above-mentioned qualitative research by quantitively assessing patient reasons for using the Eucalyptus DWLS across three countries. It is believed that the study outcomes will illuminate the importance of digital models in increasing access to continuous, MDT-guided obesity care.

## Materials and methods

Study design

To achieve its aims, the study adopted a multinational survey-based design. Investigators followed the Strengthening the Reporting of Observational Studies in Epidemiology (STROBE) Statement guidelines throughout each phase of the study. All participants provided informed consent for their de-identified data to appear in peer-reviewed research. The Bellberry Human Ethics Committee approved the study on November 22, 2023 (Approval no. 2023-05-563-A-1).

Program overview

The Eucalyptus DWLS was launched in 2021 in Australia under the brand names Juniper (for women) and Pilot (for men). Since then, it has provided care to over 100,000 PWOO across Australia, Germany, Japan, and the United Kingdom. The service is accredited by the Australian Council on Healthcare Standards [32] and the UK Digital Technology Assessment Criteria [33]. At present, the service has only ever provided medication-supported therapy, i.e., lifestyle coaching supplemented with GLP-1 RA or dual GIP/GLP-1 RA treatment, and neither component is a stand-alone offering. A Eucalyptus doctor or nurse practitioner determines patient eligibility for the service via an assessment of patient responses to an extensive pre-consultation questionnaire. These questionnaires can comprise over 100 questions and often include requests for clinical reports, blood test results, and/or photos. Eligibility decisions are based on GLP-1 RA and dual GIP/GLP-1 RA product information documents that detail body mass index (BMI) ranges, contraindications, and drug interactions [34,35]. Inclusion criteria include a BMI of 27 kg/m^2^ for patients with at least one weight-related comorbidity (e.g., dyslipidemia and hypertension) or patients of non-Caucasian ethnicity, and a BMI of 30 kg/m^2^ for Caucasian patients without any weight-related comorbidities. Key exclusion criteria included the following contraindications: a personal or family history of thyroid C-cell tumors, multiple endocrine neoplasia syndrome type 2, acute pancreatitis, acute gallbladder disease, hypoglycemia, known hypersensitivity to the chosen weight-loss medication or any of the product components, a severe mental health condition, and type 1 or type 2 diabetes.

Each eligible patient is allocated an MDT, consisting of a prescribing doctor or nurse practitioner, a university-qualified health coach (dietitian or nutritionist), a pharmacist, and a university-qualified medical support officer (nurse or pharmacist). Health coaches use patient baseline data and responses to diet- and exercise-related questions in the pre-consultation questionnaire to develop personalized lifestyle plans. Plans are forwarded to patients alongside multimodal educational materials, such as exercise videos, recipes, and macro-nutrient guides. Patients are sent automated reminders at fortnightly intervals to upload data to a weight tracker on the program app (computer-based platform in Germany). A compulsory follow-up consultation is held between patients and their prescribing physician after four or five months (depending on medication type) to determine whether an additional prescription should be issued. Patients can solicit advice from any MDT member as often as they like, to which they typically receive a response within 24 hours. At program commencement, medical support officers instruct patients to report any side effects that arise. Every communication between patients and their MDT (including prescription decisions and reported side effects) is automatically uploaded to the Eucalyptus central data repository on Metabase, which MDTs have complete access to facilitate care continuity. At the time of survey administration, a monthly subscription to the Eucalyptus DWLS cost between 189 (lowest Semaglutide dose) and 299 (highest Tirzepatide dose) Great British Pounds in the United Kingdom; between 280 and 389 Australian Dollars in Australia; and 356 Euros in Germany.

Procedures

Investigators developed a two-question survey based on findings from the qualitative study of the Eucalyptus Australia DWLS. The survey’s first question asked participants why they decided to use the Eucalyptus DWLS rather than an F2F alternative and allowed them to select multiple options from a list of six possible reasons. These reasons covered all five themes identified in the qualitative study of the Eucalyptus DWLS and an *other* option, which allowed patients to include an open-text response. The remaining question asked whether patients had a regular GP, as two of the five themes in the qualitative study concerned patient perceptions of their GP’s role in obesity care. The survey can be found in the Appendix. Surveys were prepared in Typeform (software-as-a-service company), which generates survey links and clean CSV spreadsheets for data analysis. Simple random sampling was used, whereby survey links were emailed to 1,000 active Eucalyptus DWLS patients in each of the three countries (Australia, Germany, and the United Kingdom) on April 22, 2024. This number (1,000) was selected based on outcomes from a power analysis that used 10 as its difference (standard deviation) value (90% power; alpha = 0.05). The analysis revealed that 385 respondents would suffice for statistical power. However, investigators were informed by Eucalyptus that the program's average survey response rate was just over 40%, so they divided 385 by 0.40 and rounded the resultant figure of 963 up to 1,000. 

Endpoints

The study’s co-primary endpoints were the percentage distribution of reasons for using the Eucalyptus DWLS across the total cohort and for individual countries. The effect of patient demographics and regular GP status on reasons for using the service represented the study’s secondary endpoints. 

Statistical analysis

Descriptive statistics were presented as percentage distributions and number of occurrences. As patients could select multiple reasons for subscribing to the Eucalyptus DWLS in the survey, these data were assessed as binary categorical variables (reason X did/did not influence decision). Consequently, chi-square tests were conducted to assess between-country differences, and multivariate binary logistic regression analyses were used to assess the effect of multiple predictor variables on each subscription reason while controlling for confounders. All statistical analyses were performed using RStudio, version 2023.06.1+524 (RStudio: Integrated Development Environment for R, Boston, MA).

## Results

A total of 3,000 Eucalyptus DWLS patients received the email link on April 22, 2024, of whom 1,283 (42.77%) completed the survey. This included 481 (37.49%) patients from the United Kingdom, 428 (33.36%) from Germany, and 374 (29.15%) from Australia (Table 1). The mean age was 43.26 (±14.84) years, and the mean BMI was 35.54 (± 4.85) kg/m^2^. Roughly four-fifths of participants (*N* = 1021, 79.58%) were of Caucasian heritage and 960 (74.82%) were female.

**Table 1:** Baseline characteristics.

Demographic information	
Age, Mean (±SD)	43.26 (±14.84) years
Gender, number (%)	
Female	960 (74.82)
Male	323 (25.18)
Nationality, number (%)	
United Kingdom	481 (37.49)
Germany	428 (33.36)
Australia	374 (29.15)
Ethnicity, number (%)	
Caucasian	1,021 (79.58)
Asian including subcontinent	106 (8.26)
Middle Eastern	62 (4.83)
Black African of African Caribbean	36 (2.80)
Latino/Hispanic	33 (2.57)
Rather not say	25 (1.95)
Clinical information, Mean (±SD)	
BMI	35.54 (±4.85) kg/m^2^
Weight	100.43 (±16.22) kg

Personal discomfort in discussing weight loss in F2F settings was the most common reason for subscribing to the Eucalyptus DWLS across the full cohort (*N* = 557, 43.41%) and each national cohort (Table 2). The service’s flexibility was the second most common full-cohort reason for subscription (*N* = 441, 34.37%), followed by an inability to access comprehensive obesity care through a local GP (*N* = 435, 33.90%), and marketing or brand awareness (*N* = 358, 27.90%). In total, 79 (6.16%) patients selected *other* and added an open-text response. However, after using the Braun and Clarke thematic analysis method, all these responses were re-allocated to existing response items (36 - Marketing/brand awareness; 21 - Flexibility of digital asynchronous consults; 13 - Better perceived care coordination; 9 - Uncomfortable discussing weight in F2F consultations). Chi-square tests found a significant association between patient nationality and three of the subscription reasons: marketing or brand awareness (X2(2, *N* = 1,283) = 7.85, *P* = 0.02), perception of better care coordination (X2(2, *N* = 1,283) = 7.71, *P* = 0.02), and the inaccessibility of comprehensive obesity care through a local F2F clinic (X2(2, *N* = 1,283) = 14.04, *P* < 0.01). German patients were significantly more likely to cite the latter two reasons, whereas a higher proportion UK patients indicated their subscription decision was influenced by marketing or brand awareness.

**Table 2:** Reasons for subscribing to Eucalyptus instead of F2F alternative by country. F2F, face-to-face

Reason	Country, number (%)			
	Australia	United Kingdom	Germany	Total
Marketing/brand awareness	100 (26.74)	155 (32.22)	103 (24.07)	358 (27.90)
Uncomfortable discussing weight in F2F consultation	169 (45.19)	213 (44.28)	175 (40.88)	557 (43.41)
Failure of previous stand-alone lifestyle interventions	78 (20.86)	116 (24.12)	77 (17.99)	271 (21.12)
Better perceived care coordination	50 (13.37)	55 (11.43)	76 (17.75)	181 (14.10)
Comprehensive obesity care inaccessible through F2F	112 (29.95)	148 (30.77)	175 (40.88)	435 (33.90)
Flexibility of digital asynchronous consultations	122 (32.62)	173 (35.97)	146 (34.11)	441 (34.37)

Multivariate binary logistic regression analyses were then conducted on each subscription reason to assess their association with demographic data (such as country of residence) while controlling for confounders. The first model revealed that, controlling for all other variables, UK patients were over 50% more likely to have been influenced by Eucalyptus marketing and brand awareness in their decision to subscribe to the DWLS than German patients (Table 3). Discomfort in discussing weight in F2F settings was significantly more likely to be cited as a subscription reason in non-Caucasian patients (adjusted odds ratio [aOR] = 2.288, 95% confidence interval [CI] = 1.467-3.407, *P* < 0.001), patients who had a regular GP (aOR = 0.163, 95% CI = 0.086-1.537, *P* = 0.034), and patients from the highest BMI category, compared to the lowest BMI category (aOR = 1.676, 95% CI = 1.167-2.415, *P* = 0.005) (Table 4). Gender (male) was associated with decreased odds of a patient’s subscription decision being influenced by the failure of a previous standalone lifestyle intervention (aOR = 0.333, 95% CI = 0.220-0.491, *P* = 0.001), while both Australian and UK patients were over 50% more likely to select this reason than German patients (Table 5). The fourth model found that non-Caucasian patients were statistically more likely to subscribe to Eucalyptus as a result of the perception that the service offered better care coordination than F2F alternatives (aOR = 1.594, 95% CI = 1.100-2.282, *P* = 0.012), whereas UK patients were less likely to cite this reasons than German patients (aOR = 0.609, 95% CI = 0.414-0.892, *P* = 0.011) (Table 6). Decreased odds of being influenced by the inability to access comprehensive obesity care at a local F2F clinic was observed in males (aOR = 0.710, 95% CI = 0.526-0.955, *P* = 0.025), non-Caucasian patients (aOR = 0.684, 95% CI = 0.503-0.924, *P* = 0.014), British patients (relative to German patients) (aOR = 0.653, 95% CI = 0.493-0.863, *P* = 0.003), and patients without a regular GP (aOR = 0.682, 95% CI = 0.339-1.354, *P* = 0.027) (Table 7). And finally, all else equal, female patients were over 40% more likely than males to consider the flexibility of digital asynchronous consultations as a key reason for subscribing to the Eucalyptus DWLS (Table 8).

**Table 3:** Logistic regression model of predictors of Eucalyptus DWLS subscription reason - marketing or brand awareness. *P-value < 0.01. DWLS, digital weight-loss services; GP, general practitioner; BMI, body mass index

Covariate	N	Odds ratio	95% confidence interval	*P*-value
Age	1,283	0.999	(0.990, 1.007)	0.750
Ethnicity				
Caucasian	1,021	Reference	Reference	Reference
Non-Caucasian	262	1.10	(0.809, 1.485)	0.540
Gender				
Female	960	Reference	Reference	Reference
Male	323	1.236	(0.918, 1.658)	0.160
BMI category				
27.5-29.99 kg/m^2^	238	Reference	Reference	Reference
30-34.99 kg/m^2^	386	1.251	(0.862, 1.827)	0.242
35-39.99 kg/m^2^	388	1.278	(0.883, 1.863)	0.20
≥40 kg/m^2^	271	1.401	(0.945, 2.087)	0.095
Country				
Germany	428	Reference	Reference	Reference
United Kingdom	481	1.502	(1.117, 2.026)	0.007*
Australia	374	1.064	(0.756, 1.495)	0.721
GP status				
Had a regular GP	1025	Reference	Reference	Reference
No regular GP	215	1.023	(0.727, 1.425)	0.90
Unsure	43	1.127	(0.558, 2.159)	0.727

**Table 4:** Logistic regression model of predictors of Eucalyptus DWLS subscription reason - patient discomfort discussing weight in F2F consultations. *P-value < 0.001 **P-value < 0.01. ***P-value < 0.05. DWLS, digital weight-loss services; GP, general practitioner; F2F, face-to-face; BMI, body mass index

Covariate	N	Odds ratio	95% confidence interval	*P*-value
Age	1,283	0.995	(0.987, 1.002)	0.170
Ethnicity				
Caucasian	1,021	Reference	Reference	Reference
Non-Caucasian	262	3.288	(2.467, 4.407)	<0.001*
Gender				
Female	960	Reference	Reference	Reference
Male	323	1.166	(0.881, 1.544)	0.282
BMI category				
27.5-29.99 kg/m^2^	238	Reference	Reference	Reference
30-34.99 kg/m^2^	386	1.055	(0.750, 1.487)	0.760
35-39.99 kg/m^2^	388	0.998	(0.710, 1.406)	0.991
≥40 kg/m^2^	271	1.676	(1.167, 2.415)	0.005**
Country				
Germany	428	Reference	Reference	Reference
United Kingdom	481	1.164	(0.882, 1.537)	0.285
Australia	374	1.075	(0.788, 1.468)	0.647
GP status				
Had a regular GP	1025	Reference	Reference	Reference
No regular GP	215	0.104	(0.086, 0.128)	0.034***
Unsure	43	0.163	(0.131, 0.204)	0.157

**Table 5:** Logistic regression model of predictors of Eucalyptus DWLS subscription reason - failure of previous standalone lifestyle intervention. *P-value < 0.001. **P-value < 0.05. ***P-value < 0.01. DWLS, digital weight-loss services; GP, general practitioner; BMI, body mass index

Covariate	N	Odds ratio	95% confidence interval	*P*-value
Age	1,283	1.005	(0.989, 1.011)	0.30
Ethnicity				
Caucasian	1021	Reference	Reference	Reference
Non-Caucasian	262	0.816	(0.566, 1.158)	0.264
Gender				
Female	960	Reference	Reference	Reference
Male	323	0.333	(0.220, 0.491)	<0.001*
BMI category				
27.5-29.99 kg/m^2^	238	Reference	Reference	Reference
30-34.99 kg/m^2^	386	0.048	(0.691, 1.606)	0.821
35-39.99 kg/m^2^	388	0.396	(0.993, 2.245)	0.057
≥40 kg/m^2^	271	1.133	(0.728, 1.770)	0.581
Country				
Germany	428	Reference	Reference	Reference
United Kingdom	481	1.525	(1.095, 2.131)	0.013**
Australia	374	1.652	(1.134, 2.409)	0.009***
GP status				
Had a regular GP	1025	Reference	Reference	Reference
No regular GP	215	0.961	(0.649, 1.400)	0.840
Unsure	43	1.615	(0.803, 3.113)	0.162

**Table 6:** Logistic regression model of predictors of Eucalyptus DWLS subscription reason - perception of better care coordination. *P-value < 0.05. DWLS, digital weight-loss services; GP, general practitioner; BMI, body mass index

Covariate	N	Odds ratio	95% confidence interval	*P*-value
Age	1,283	1.000	(0.994, 1.010)	0.985
Ethnicity				
Caucasian	1,021	Reference	Reference	Reference
Non-Caucasian	262	1.594	(1.100, 2.282)	0.012*
Gender				
Female	960	Reference	Reference	Reference
Male	323	0.737	(0.484, 1.099)	0.145
BMI category				
27.5-29.99 kg/m^2^	238	Reference	Reference	Reference
30-34.99 kg/m^2^	386	1.327	(0.824, 2.182)	0.253
35-39.99 kg/m^2^	388	0.867	(0.523, 1.459)	0.586
≥40 kg/m^2^	271	1.548	(0.937, 2.600)	0.092
Country				
Germany	428	Reference	Reference	Reference
United Kingdom	481	0.609	(0.414, 0.892)	0.011*
Australia	374	0.751	(0.493, 1.136)	0.178
GP status				
Had a regular GP	1025	Reference	Reference	Reference
No regular GP	215	1.339	(0.867, 2.025)	0.176
Unsure	43	1.108	(0.410, 2.522)	0.822

**Table 7:** Logistic regression model of predictors of Eucalyptus DWLS subscription reason - comprehensive obesity care inaccessible at a local F2F clinic. DWLS, digital weight-loss services; GP, general practitioner; F2F, face-to-face; BMI, body mass index *P-value < 0.05. **P-value < 0.01.

Covariate	N	Odds ratio	95% confidence interval	*P*-value
Age	1,283	1.002	(0.994, 1.010)	0.557
Ethnicity				
Caucasian	1,021	Reference	Reference	Reference
Non-Caucasian	262	0.684	(0.503, 0.924)	0.014*
Gender				
Female	960	Reference	Reference	Reference
Male	323	0.710	(0.526, 0.955)	0.025*
BMI category				
27.5-29.99 kg/m^2^	238	Reference	Reference	Reference
30-34.99 kg/m^2^	386	0.926	(0.656, 1.309)	0.663
35-39.99 kg/m^2^	388	0.911	(0.646, 1.288)	0.596
≥40 kg/m^2^	271	0.796	(0.547, 1.158)	0.236
Country				
Germany	428	Reference	Reference	Reference
United Kingdom	481	0.653	(0.493, 0.863)	0.003**
Australia	374	0.736	(0.537, 1.007)	0.056
GP status				
Had a regular GP	1025	Reference	Reference	Reference
No regular GP	215	0.682	(0.483, 0.952)	0.027*
Unsure	43	0.699	(0.339, 1.354)	0.307

**Table 8:** Logistic regression model of predictors of Eucalyptus DWLS subscription reason - flexibility of digital asynchronous consultations. DWLS, digital weight-loss services; GP, general practitioner; F2F, face-to-face; BMI, body mass index *P-value < 0.001.

Covariate	N	Odds ratio	95% confidence interval	*P*-value
Age	1,283	1.001	(0.993, 1.008)	0.877
Ethnicity				
Caucasian	1,021	Reference	Reference	Reference
Non-Caucasian	262	0.932	(0.693, 1.246)	0.637
Gender				
Female	960	Reference	Reference	Reference
Male	323	0.576	(0.426, 0.775)	<0.001*
BMI category				
27.5-29.99 kg/m^2^	238	Reference	Reference	Reference
30-34.99 kg/m^2^	386	1.140	(0.806, 1.619)	0.462
35-39.99 kg/m^2^	388	1.023	(0.722, 1.454)	0.898
≥40 kg/m^2^	271	1.340	(0.928, 1.942)	0.120
Country				
Germany	428	Reference	Reference	Reference
United Kingdom	481	1.086	(0.821, 1.436)	0.565
Australia	374	1.049	(0.764, 1.439)	0.769
GP status				
Had a regular GP	1025	Reference	Reference	Reference
No regular GP	215	1.345	(0.980, 1.841)	0.065
Unsure	43	1.272	(0.666, 2.371)	0.454

## Discussion

To the knowledge of the authors, this is the first study to quantitatively assess patient reasons for subscribing to a real-world, medication-supported DWLS. While previous literature has indicated that digital services can play a pivotal role in increasing access to chronic MDT care [14,15,16,17] various influential stakeholders have questioned whether medication-supported DWLSs are simply being used by patients to facilitate access to weight-loss medications rather than treating the medications as a supplement to lifestyle therapy [9,18]. Findings from a 2024 qualitative study aligned with both arguments [11]. This study reinforced those findings with quantitative data across multiple countries and enriched them with important nuances.

Arguably, the most interesting discovery was that patient discomfort in discussing their weight in F2F consultations was the most common reason for subscribing to the Eucalyptus DWLS instead of F2F alternatives. Perceived stigma has been identified as a common barrier to F2F care across several chronic diseases, including sexual and mental health [12,32]. While it was also identified as a key theme in the qualitative study of the Eucalyptus DWLS [11], the finding in this analysis that over forty percent of the entire cohort and each country cited it as a factor behind their subscription illuminates the extent of the factor’s significance as a barrier to F2F obesity care. Moreover, the discovery that non-Caucasian patients were 128 percent more likely to cite this reason for subscribing to Eucalyptus suggests that this barrier may be compounded by perceptions of ethnic stigma among patients with ethnic minority backgrounds. It is also worth highlighting that patients from the highest BMI category were overrepresented in this subscription reason.

The study also generated several other interesting findings. Over a third of the full cohort indicated that the flexibility of the Eucalyptus DWLS’ asynchronous consultations contributed to their subscription. This discovery is consistent with other literature on the temporal benefits of digital modalities in chronic care settings and adds support to the arguably understated argument about the difficulty of managing ongoing MDT consults with significant work and family commitments in modern society [15,17,33]. The fact that female patients were significantly more likely to cite this as a factor in their Eucalyptus subscription may reflect the increasing normalization of full-time female employment during early parenthood, without commensurate reallocation of domestic duties to male partners in heterosexual relationships. Interestingly, females were also statistically more likely than males to cite the failure of previous standalone interventions and an inability to access comprehensive obesity care at local F2F services as reasons for their subscription. The former could be a reflection of the added difficulty of trying to lose weight during and after menopause [36]. The gender disparity in inability to access comprehensive obesity care through F2F services may be attributable to women’s better understanding of such care, as previous studies have found health literacy to be higher among women [37]. It is also important to note that these latter two reasons (failure of standalone, and inaccessibility through F2F) may also reflect patients’ desire to obtain weight-loss medications and perhaps prioritize them in their care journey. Roughly a quarter to a third of patients in each country cited marketing and brand awareness as a reason behind their subscription. It is possible that a significant number of these patients were attracted by the service’s weight-loss medications given the strong focus on medications in the company’s marketing content [30].

Limitations

The study had several limitations. First, the study survey allowed patients to select multiple responses and thus results may have been affected by secondary reasons for subscribing to the program. Second, the survey did not include a question that specifically asked patients whether they had subscribed to the DWLS primarily to access weight-loss medications, and therefore, this key stakeholder concern about medicated DWLSs could not be directly addressed. The reason for omitting such a question was that the investigators believed the only reasonable way of assessing this possibility would be to measure multiple markers of patient-MDT engagement rather than soliciting survey responses. Thirdly, the Eucalyptus DWLS is relatively expensive in all three of the studied populations and is likely inaccessible to patients from lower socioeconomic groups. Finally, the study sample included a disproportionate number of female and Caucasian patients and thus did not reflect the diversity of Australian, German, and British populations. 

Future research

Future investigations should build on the findings of this study by quantitatively assessing patient-MDT messaging frequency and health coaching platform engagement in a cohort of patients from a medication-supported DWLS such as Eucalyptus. Researchers should also consider a deeper exploration of the potential intersectionality of high BMI and ethnicity in the stigmatization of F2F obesity care, along with a focused analysis of the possible disproportionate temporal burden on females trying to manage ongoing MDT obesity consultations in F2F settings.

## Conclusions

This study contributed foundational knowledge on the utility of medication-supported DWLSs and added another essential layer to the budding literature on these services. Against the backdrop of the obesity epidemic and the promising outcomes emerging from medication-supported weight-loss interventions, it is arguably unsurprising that an increasingly large number of people are subscribing to these services. However, despite general consensus around the access benefits of digital modalities across a range of interventions (especially those for chronic diseases that require ongoing MDT care), ongoing debates about the net utility of medication-supported DWLSs have tended to overlook access-related arguments. This study extended upon the findings of a previous qualitative study of the Eucalyptus Australia DWLS by quantitatively assessing the reasons patients subscribe to the service in three of its operating countries. The study found that patient discomfort in discussing their weight in F2F consultations was the most common reason for subscribing to the Eucalyptus DWLS across the full cohort (*N* = 557, 43.41%) and each national cohort (all >40%). Non-Caucasian patients were 128% more likely to select this reason. The DWLS model's flexibility (*N* = 441, 34.37%) and patients' perceived inability to access comprehensive obesity care through a local GP (*N* = 435, 34.37%) were also key factors behind their Eucalyptus subscription. However, consistent with the previous qualitative study, investigators also found that a significant portion of patients used the program for reasons that suggest that they are primarily interested in accessing weight-loss medications. Specifically, over a quarter of patients (*N* = 358, 27.90%) cited marketing and brand awareness as their main subscription reason, and over a fifth cited failure of previous lifestyle interventions (*N* = 271, 21.12%). Further research is needed to ascertain whether patients of this nature are properly engaging with the (core) health coaching component of DWLSs like Eucalyptus. 

## Appendices

Appendix: Patient survey

Question 1: Why did you decide to use Eucalyptus to treat your weight condition, rather than seeking care from traditional face-to-face (F2F) services?

-               I feel more comfortable receiving weight-loss counseling online than through F2F services

-               I love the flexibility of online asynchronous consults

-               Coordinated consults and information between multiple F2F clinicians is too difficult

-               My local F2F clinics don’t offer comprehensive weight-loss treatment

-               I was attracted by the company’s marketing material/I had heard of the brand before

-               Other (please specify)

Question 2: Do you currently have a regular GP? (A regular GP is a GP you have an ongoing relationship with, i.e., a GP you tend to consult whenever you have a health issue.)

-               Yes

-               No

-               Unsure

## References

[1] (2024). Global incidence, prevalence, years lived with disability (YLDs), disability-adjusted life-years (DALYs), and healthy life expectancy (HALE) for 371 diseases and injuries in 204 countries and territories and 811 subnational locations, 1990-2021: a systematic analysis for the Global Burden of Disease Study 2021. Lancet.

[2] (2024). World Health Organization. Obesity and Overweight. March.

[3] Hall KD, Heymsfield SB, Kemnitz JW, Klein S, Schoeller DA, Speakman JR (2012). Energy balance and its components: implications for body weight regulation. Am J Clin Nutr.

[4] Javed Z, Valero-Elizondo J, Maqsood MH (2022). Social determinants of health and obesity: findings from a national study of US adults. Obesity (Silver Spring).

[5] Wang K, Wu C, Yao Y, Zhang S, Xie Y, Shi K, Yuan Z (2022). Association between socio-economic factors and the risk of overweight and obesity among Chinese adults: a retrospective cross-sectional study from the China Health and Nutrition Survey. Glob Health Res Policy.

[6] Verde L, Frias-Toral E, Cardenas D (2023). Editorial: Environmental factors implicated in obesity. Front Nutr.

[7] (2024). World Health Organization. Health Service Framework for Prevention and Management of Obesity. Geneva.

[8] (2023). National Institute for Health and Care Excellence. Semaglutide for Managing Overweight and Obesity. Semaglutide for managing overweight and obesity, September 2023 (accessed on 17 August 2024).

[9] Golovaty I, Hagan S (2024). Direct-to-consumer platforms for new antiobesity medications—Concerns and potential opportunities. N Engl J Med.

[10] Irvin L, Madden LA, Marshall P, Vince RV (2023). Digital health solutions for weight loss and obesity: a narrative review. Nutrients.

[11] Talay L, Vickers M, Loftus S (2024). Why people with overweight and obesity are seeking care through digital obesity services: a qualitative analysis of patients from Australia’s largest digital obesity provider. Telemed Rep.

[12] Naslund JA, Deng D (2021). Addressing mental health stigma in low-income and middle-income countries: a new frontier for digital mental health. Ethics Med Public Health.

[13] Song HJ, Dennis S, Levesque JF, Harris MF (2019). What matters to people with chronic conditions when accessing care in Australian general practice? A qualitative study of patient, carer, and provider perspectives. BMC Fam Pract.

[14] Phillip M, Bergenstal RM, Close KL (2021). The digital/virtual diabetes clinic: the future is now-recommendations from an international panel on diabetes digital technologies introduction. Diabetes Technol Ther.

[15] Georgieva N, Tenev V, Kamusheva M (2023). Diabetes mellitus - digital solutions to improve medication adherence: scoping review. Diabetology.

[16] Forbes A, Keleher MR, Venditto M, DiBiasi F (2023). Assessing patient adherence to and engagement with digital interventions for depression in clinical trials: systematic literature review. J Med Internet Res.

[17] Torous J, Jän Myrick K, Rauseo-Ricupero N, Firth J (2020). Digital mental health and COVID-19: using technology today to accelerate the curve on access and quality tomorrow. JMIR Ment Health.

[18] Payne Payne, H. No Doctor (2024). No Script, No Worries: Here’s Your Ozempic. https://www.medicalrepublic.com.au/no-doctor-no-script-no-worries-heres-your-ozempic/107426.

[19] Wilding J, Batterham R, Calanna S (2021). Once-weekly Semaglutide in adults with overweight or obesity. N Engl J Med.

[20] Wadden TA, Bailey TS, Billings LK (2021). Effect of subcutaneous Semaglutide vs placebo as an adjunct to intensive behavioural therapy on body weight in adults with overweight or obesity: the STEP 3 randomized clinical trial. JAMA.

[21] Jastreboff AM, Aronne LJ, Ahmad NN (2022). Tirzepatide once weekly for the treatment of obesity. N Engl J Med.

[22] Yabe D, Kawamori D, Seino Y, Oura T, Takeuchi M (2023). Change in pharmacodynamic variables following once-weekly tirzepatide treatment versus dulaglutide in Japanese patients with type 2 diabetes (SURPASS J-mono substudy). Diabetes Obes Metab.

[23] Linge J, Neelan IJ, Dahlqvist Leinhard O (2023). Tirzepatide Achieves Significant Weight Loss Without Adverse Effects on Muscle Composition (SURPASS-3). https://www.easd.org/media-centre/home.html?j=118422&sfmc_sub=77326945&l=327_HTML&u=1738265&mid=110007893&jb=1#!resources/tirzepatide-achieves-significant-weight-loss-without-adverse-effects-on-muscle-composition-surpass-3-mri-d33f2a13-d83e-4112-8763-621e82d3e128.

[24] Talay L, Vickers M (2024). Effectiveness and side effect incidence in a real-world digital weight-loss service using compounded Semagltuide: a retrospective comparative study. Obesities.

[25] Talay L, Vickers M, Ruiz L (2024). Effectiveness of an email-based, Semaglutide-supported weight-loss service for people with overweight and obesity in Germany: a real-world retrospective cohort analysis. Obesities.

[26] Talay L, Vickers M (2024). Effectiveness and care continuity in an app-based, glucagon-like peptide-1 receptor agonist-supported weight-loss service for women with overweight and obesity in the UK: a real-world retrospective cohort analysis. Diabetes Obes Metab.

[27] Talay L, Vickers M, Fuller S (2024). GLP-1 RA prescribing errors in a multidisciplinary digital weight-loss service: a retrospective quantitative analysis. Healthcare (Basel).

[28] Talay L, Vickers M (2024). The dispensing error rate in an app-based, semaglutide-supported weight-loss service: a retrospective cohort study. Pharmacy (Basel).

[29] Talay L, Vickers M (2024). Patient adherence to a real-world Digital, asynchronous weight loss program in Australia that combines behavioural and GLP-1 RA therapy: a mixed methods study. Behav Sci (Basel).

[30] Australian Council on Healthcare Standards (accessed on 8 August 2024) (2024). Australian Council on Healthcare Standards. ACHS International.

[31] (2024). Digital Technology Assessment Criteria. NHS England. https://transform.england.nhs.uk/key-tools-and-info/digital-technology-assessment-criteria-dtac/ .

[32] Valentine JA, Delgado LF, Haderxhanaj LT, Hogben M (2022). Improving sexual health in U.S. rural communities: reducing the impact of stigma. AIDS Behav.

[33] Fernandes LG, Devan H, Fioratti I, Kamper SJ, Williams CM, Saragiotto BT (2022). At my own pace, space, and place: a systematic review of qualitative studies of enablers and barriers to telehealth interventions for people with chronic pain. Pain.

[34] (2024). Wegovy Semaglutide Injection: Prescribing Information. Novo Nordisk, 2024. Bagsvaerd, Denmark. https://www.novo-pi.com/wegovy.pdf.

[35] (2024). Mounjaro Tirzepatide Injection: Product Monograph Including Patient Medication Information. https://pi.lilly.com/ca/mounjaro-ca-pm.pdf.

[36] Simpson SJ, Raubenheimer D, Black KI, Conigrave AD (2023). Weight gain during the menopause transition: evidence for a mechanism dependent on protein leverage. BJOG.

[37] Zhang F, Or PP, Chung JW (2021). How different health literacy dimensions influences health and well-being among men and women: The mediating role of health behaviours. Health Expect.

